# A Universal Study on the Effect Thermal Imidization Has on the Physico-Chemical, Mechanical, Thermal and Electrical Properties of Polyimide for Integrated Electronics Applications

**DOI:** 10.3390/polym14091713

**Published:** 2022-04-22

**Authors:** Imadeddine Benfridja, Sombel Diaham, Fathima Laffir, Grace Brennan, Ning Liu, Tadhg Kennedy

**Affiliations:** 1 Department of Chemical Sciences, University of Limerick, Limerick V94 T9PX, Ireland; imadeddine.benfridja@ul.ie; 2 Bernal Institute, University of Limerick, Limerick V94 T9PX, Ireland; fathima.laffir@ul.ie (F.L.); gracebrennan11@gmail.com (G.B.); ning.liu@ul.ie (N.L.); 3 LAPLACE Institute, University of Toulouse, Université Paul Sabatier, 31062 Toulouse, France; sombel.diaham@laplace.univ-tlse.fr; 4 Department of Physics, University of Limerick, Limerick V94 T9PX, Ireland

**Keywords:** polyimide, imidization, thermal curing

## Abstract

Polyimides (PI) are a class of dielectric polymer used in a wide range of electronics and electrical engineering applications from low-voltage microelectronics to high voltage isolation. They are well appreciated because of their excellent thermal, electrical, and mechanical properties, each of which need to be optimized uniquely depending on the end application. For example, for high-voltage applications, the final polymer breakdown field and dielectric properties must be optimized, both of which are dependent on the curing process and the final physico-chemical properties of PI. The majority of studies to date have focused on a limited set of properties of the polymer and have analyzed the effect of curing from a physicochemical-, mechanical- or electrical-centric viewpoint. This paper seeks to overcome this, unifying all of these characterizations in the same study to accurately describe the universal effect of the cure temperature on the properties of PI and at an industrial processing scale. This paper reports the widest-ranging study of its kind on the effect that cure temperature has on the physico-chemical, mechanical, thermal and electrical properties of polyimide, specifically poly (pyromellitic dianhydride-co-4, 4′-oxydianiline) (PMDA/ODA). The optimization of the cure temperature is accurately studied not only regarding the degree of imidization (DOI), but also considering the entire physical properties. Particularly, the analysis elucidates the key role of the charge–transfer complex (CTC) on these properties. The results show that while the thermal and mechanical properties improve with both DOI and CTC formation, the electrical properties, particularly at high field conditions, show an antagonistic behavior enhancing with increasing DOI while degrading at higher temperatures as the CTC formation increases. The electrical characterization at low field presents an enhancement of the final PI properties likely due to the DOI. On the contrary, at high electric field, the conductivity results show an improvement at an intermediate temperature emphasizing an ideal compromise between a high DOI and PI chain packing when the thermal imidization process is performed over this equilibrium. This balance enables maximum performance to be obtained for the PI film with optimized electrical properties and, overall, optimal thermal and mechanical properties are achieved.

## 1. Introduction

Polyimides (PIs) are a broad class of polymer used in a variety of applications such as photovoltaics, aerospace engineering and electronics. For example, in the electronics industry, they are widely used as a dielectric material for high temperature capacitors, bond pad redistribution layers, stress buffers for enhanced mechanical properties, surface passivation for power devices and wafer-layer packaging. Aromatic polyimides have been extensively used in this regard due to their thermal stability (>500 °C), high glass transition temperature (T_g_), mechanical toughness, chemical resistance, excellent dielectric properties and inherently low coefficient of thermal expansion (CTE) [1].

PI synthesis was first described in 1955 and occurs via a two-step process [2,3]. In the first step, a polyamic acid (PAA) solution which is the precursor of polyimide is prepared. PAA is synthesized via the reaction between two precursor monomers (i.e., a dianhydride and a diamine) at room temperature in polar aprotic solvents such as *N*-methyl-2-pyrrolidone (NMP), *N*,*N*-dimethylformamide (DMF) or *N*,*N*-dimethylacetamide (DMAc). Many varieties of PAA can be synthesized leading to hundreds of different polyimides (e.g., poly (4,4′-oxydiphenylene pyromellitimide) (PMDA-ODA), poly (4-4′-oxydiphenylene biphenyltetracarboximide) (BPDA-ODA), poly (p-phenylene oxydiphthalimide) (ODPA-PDA), etc.). In the second step, PAA is cyclodehydrated using a thermal or chemical conversion process to form the final insoluble and infusible polyimide. PMDA-ODA polyimides have been widely investigated due to their synthetic simplicity, outstanding solvent resistance, high temperature stability (>400 °C) and high tensile properties [4].

The final physical properties of PIs and their integrity during ageing depend strongly on the control and optimization of the curing process. Two concomitant transformations occur during curing, namely imidization (i.e., the conversion of amic acid groups in PAA into an imide), and charge–transfer complex (CTC) formation. The formation of CTCs between the dianhydride (acceptor) and diamine groups (donor) was first proposed by Kotov in 1977 [5]. Based on the spectral absorption shift in the UV–visible range, it has been proposed that aromatic polyimide chains interact with each other via charge transfer or electronic polarization. This transfer remains different from a crystallization structuration that can occur in thermoplastic polymers, as CTC occurs in amorphous materials such as PI.

In recent years, many researchers have been interested in investigating the optimization of processing conditions for the imidization reaction. The chemical phenomena taking place during the imidization of PAA films merit special attention. It has been reported that the degree of imidization (DOI) (i.e., the normalized conversion ratio between PAA and imide groups) and the molecular packing of the imidized PI film are functions of the cure temperature and time [6,7]. In fact, the physico-chemical, mechanical and electrical properties of PI depend heavily on the optimization of the curing procedure, with this processing step being crucial for industrial applications where precise control of material properties is required. Moreover, such an optimum can vary between application areas depending on what are the most important properties required. For example, applications in optics, microelectronics or high-voltage electronics will potentially not all have the same requirements in terms of curing optimization. To date, a large number of studies have been reported on the effects of cure temperature on the imidization reaction (cf. Table 1). The majority of these have focused on a limited set of properties of the polymer and have analyzed the material from a physicochemical-, mechanical- or electrical-centric viewpoint. Only a very low number have tried to correlate several physical properties, but none of them have unified all these characterizations in the same study to accurately describe the universal effect of the cure temperature on the physico-chemical, thermal, mechanical and electrical properties of PI and at industrial processing scale. As such, a more unified approach is required.

In this paper, a complete characterization of PMDA-ODA polyimide under various curing temperatures (200 °C to 380 °C) was performed. A universal approach, analyzing the cure temperature’s effect on a wide range of parameters including all relevant chemical (i.e., DOI, CTC formation, binding energy), thermal (T_g_, thermal stability), mechanical (i.e., tensile strength, Young’s modulus, loss factor, CTE) and electrical properties (i.e., permittivity, dielectric loss factor, DC conductivity, AC breakdown field), has been used to unify understanding. This, to the best of our knowledge, is the most wide-ranging study of its kind. It has been shown using FTIR that the maximum degree of imidization occurs at 320 °C. Additional analysis using XPS, UV–Vis spectroscopy and profilometry shows that the extent of charge–transfer complex formation has an impact on the chemical, and optical properties of the films, whereas the mechanical and electrical properties present an enhancement at a higher curing temperature.

## 2. Materials and Methods

To assess the effect that cure temperature has on the physico-chemical properties of polyimide, samples were synthesized from polyamic acid (PAA) precursor solution consisting of pyromellitic dianhydride (PMDA) and 4,4′-oxydianiline (ODA) in a *N*-Methyl-2-pyrrolidone (NMP) solvent. The precursor has a density of 1.1 g/cm^3^, viscosity of 25–35 poise at 23 °C, 45–55 wt.% of NMP and 25–35 wt.% polyamic acid. PAA was spin-coated onto silicon wafer substrates; spin speed was adjusted to yield PI films with thickness of approx. 20 μm after curing over a range of temperatures from 200 °C up to 380 °C in steps of 20 °C. Attenuated total reflectance Fourier transform infrared (ATR-FTIR) spectroscopy was performed using a Cary 630 FTIR spectrometer (Agilent, Santa Clara, CA, USA). An average of 128 scans were collected for each sample ranging from 600 to 4000 cm^−1^. These scans were baseline-corrected as all spectra have been normalized to the typical 1500 cm^−1^ C=C absorption band. For each sample, the average value of twelve measurements was considered. X-ray photoelectron spectroscopy (XPS) was carried out using a Kratos ULTRA spectrometer (Kratos AXIS Ultra, Manchester, UK) using monochromatic Al Ka 1486.58 eV. The C 1s line at 284.8 eV was used as charge reference to determine the core level binding energies. The pass energy 1 eV was used for the survey spectra and 0.05 eV for the narrow region. Ultraviolet-visible spectroscopy (UV–Vis) was carried out on a Cary 4000 UV–Vis spectrophotometer (Agilent, Santa Clara, CA, USA). Samples were analyzed in transmission mode between 200 and 900 nm. The X-ray diffraction (XRD) was recorded at room temperature (Panalytical Empyrean, Malvern, UK). XRD data were collected for continuous scan over a range of 2θ from 5° to 70°. Differential scanning calorimetry (DSC, Netzsch, Selb, Germany) analysis was recorded using a Netzsch Polyma 214 thermal analysis system from 20 °C up to 480 °C in nitrogen at a heating rate of 20 °C/min. For the measurements, 5 to 10 mg of PI films have been used. Moreover, thermogravimetric analysis (TGA) was performed on a Perkin Elmer TGA 4000 analyzer (Perkinelmer, Waltham, MA, USA) from 30 to 400 °C in nitrogen at a heating rate of 10 °C/min. In addition, to obtain the temperature dependency of the Young’s storage modulus (E’) and the mechanical loss factor (tanδ_m_), dynamic mechanical thermal analysis (DMTA) was performed on a TA Instruments Q8000 thermal analysis system (TA Instruments, New Castle, DE, USA). The temperature was ramped from 35 to 400 °C at a heating rate of 3 °C/min and at a frequency of 1 Hz. In addition, tensile strength testing was carried out on a CellScale Biotester, where samples have been analyzed in the uni-axial mode with a displacement of 0.5 mm. PL measurements were carried out at room temperature: PL was collected by a spectrograph (Andor Kymera193i, Oxford Instrument, Oxford, UK) equipped with a visible CCD (Andor Newton DU920P, Oxford Instrument, Oxford, UK), the excitation light source was 405 nm, laser diode (Thorlabs CPS405, Thorlabs, Newton, NJ, USA).

Three techniques were employed for the electrical characterization: broadband dielectric relaxation spectroscopy (BDRS), conduction current (CC) and breakdown voltage tests (BD). For all the electrical tests, the samples were dried in oven at 150 °C for 2 days to remove any moisture influence. BDRS has been performed using a Novocontrol Alpha-A spectrometer (Novocontrol Technologies, Montabaur, Germany) from 10−1 to 106 Hz measuring the dielectric permittivity (ε’), the loss factor (tanδ_d_) and the alternating conductivity (σ_AC_) as a function of temperature between 0 and 350 °C under nitrogen. For CC, the samples were connected to a high voltage DC source and the transient current has been recorded at room temperature through a Keithley electrometer. The DC voltage was applied by consecutive steps from 500 V up to 6.5 kV for 30 min to reach the steady-state current. The DC conductivity (σ_DC_) was then calculated from each leakage current. For BD testing, each sample was sandwiched in a cell made up with a high voltage tip and a grounded plane electrode. An AC 50 Hz sine voltage using an HV amplifier with a rising ramp of 500 V/s was applied on each sample until breakdown in the range up to 10 kV_rms_. All the tests were carried out at room temperature by immersing the PI films in an insulating liquid to avoid surface flashover. A statistical analysis of the breakdown data using the Weibull law was applied considering a population of 10 samples for each PI curing condition.

## 3. Results and Discussion

**Figure 1:**
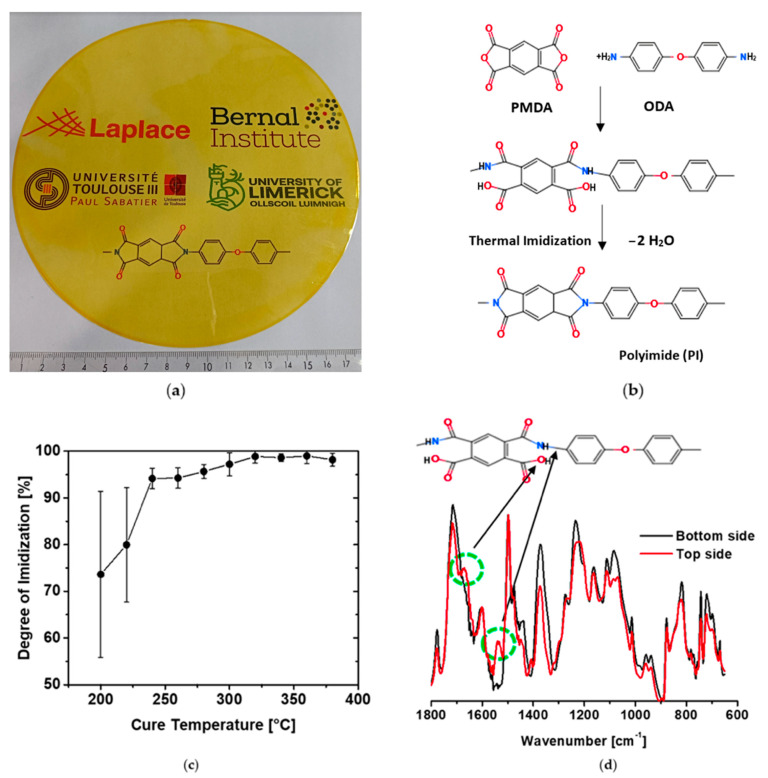
(**a**) Polyimide film peeled-off from an 8-inch wafer cured at 240 °C. (**b**) Synthesis steps of the PMDA/ODA PI. (**c**) Effect of curing temperature on the degree of imidization. (**d**) ATR-FTIR spectra for PI films cured at 200 °C (top and bottom sides).

PAA is cyclized as a result of the curing process, with the carbon atom of the amic acid group bonding to nitrogen to form an imide bond. This process, referred to as imidization (Figure 1a shows polyimide film cured at 240 °C), is accompanied by the loss of H_2_O (Figure 1b) [32]. The evaporation of both the NMP solvent and loss of H_2_O results in a decrease in the weight of the initial film, as characterized by TGA analysis up to 300 °C (Figure S1, see Supplementary Materials). The maximum rate of weight decrease is centered just above 200 °C and is related to evaporation of NMP which has a boiling point of 202 °C. A gradual and steady weight decrease is observed above this related to dehydration of the precursor.

In order to analyze the effect cure temperature has on the degree of imidization, ATR-FTIR analysis was performed. ATR-FTIR is a surface technique, therefore, to ensure the results were representative of the entire sample, twelve measurements were taken in total: six measurements from the top side and six from the bottom side of the layer (i.e., side that was directly contacted to the wafer during curing). The ATR-FTIR spectra of the PI films at each curing temperature are presented in Figure S2 in the Supplementary Materials. To determine the DOI, the ratio of the imide peak height at 1370 cm^−1^ to that at 1500 cm^−1^ was calculated at each cure temperature and normalized against the PI sample that exhibited maximum imidization according to Equation (1) [33].

The peak at 1500 cm^−1^ corresponds to the aromatic ring and was chosen as the internal standard as it remains unchanged during the imidization process. In Equation (1), A represents the height of a specific absorption peak at either 1370 cm^−1^ or 1500 cm^−1^ of the PI films cured at a given temperature. A’ represents the height of the same absorption peaks for the PI sample that demonstrated the maximal degree of imidization, which occurs at and above 320 °C.

(1) $$DOI=\frac{A_{1370}/A_{1500}}{A_{1370}^{\prime }/A_{1500}^{\prime }}$$

The average of DOI values as a function of the temperature are plotted in Figure 1c. The DOI increases with increasing cure temperature up to 320 °C, above which it reaches a plateau. There is no significant change in DOI above this temperature, with all values being within the margin of error at higher temperatures. Notably, at the two lowest cure temperatures (200 and 220 °C), the DOI was considerably different on each side of the PI film (Figure S3, see Supplementary Materials). The side directly in contact with the wafer exhibited a much higher DOI compared to the top side (89.0% and 90.4% DOI for the side contacted to the wafer and 54.1% and 67.5% for the bottom side of films cured at 200 °C and 220 °C, respectively). This is evident from the ATR-FTIR spectra in Figure 1d, which reveals two additional peaks at 1536 cm^−1^ and 1655 cm^−1^ (associated with the amide on the under-cured sides (Figure S4, see Supplementary Materials)). This discrepancy is likely related to a more efficient transfer of heat from the substrate to the side of the film in contact with the wafer compared to the top side. 

**Figure 2:**
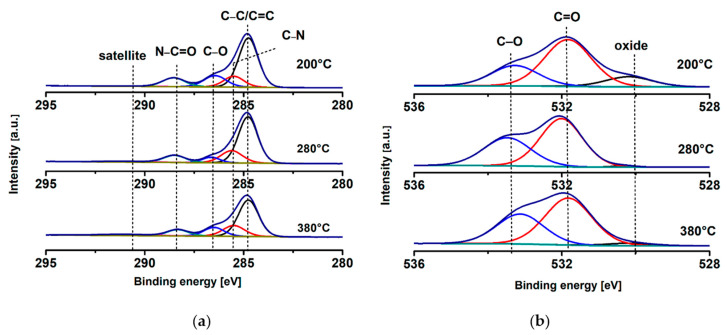
High-resolution XPS scans of (**a**) C 1s, (**b**) O 1s.

To further probe the effect of cure temperature on the chemical structure of PI, XPS was performed to identify the chemical environments present in PI films cured at 200 °C, 280 °C and 380 °C. High-resolution spectra of C 1s were deconvoluted into five peaks at binding energies ~284.8, 285.5, 286.5, 288.4 and 291 eV (Figure 2a). The peak at 284.8 eV is assigned to C–C/C=C hydrocarbon species including the aromatic rings present in the polyimide structure. The peak at 285.5 eV is assigned to C–N imide groups in the cured PI. The related nitrogen of C–N is present as a single peak at 400.2 eV in the N 1s spectra (Figure S5, see Supplementary Materials). The peak at 286.5 eV is characteristic of C–O bonding present in the ODA unit of the PI. The peak component at 288.3 eV is characteristic of the imide groups N–C=O in the cured PI. Interestingly, the absence of a peak at 289.5 eV, which is characteristic of the PDMA di-anhydride unit, O=C–O–C=O, is indicative of a high degree of imidization of PI at least on the surface of the films. The presence of the aromatic ring in the polyimide structure is further evidenced by the broad π to π* satellite peaks at 291 eV. As the curing temperature increased from 200 °C to 380 °C, the C–N component peak at 285.5 eV (Figure 2a) and the related N peak at 400.2 eV (Figure S6, Supplementary Materials) were both observed to increase from 9.6 at.% to 13.7 at.% and 2.1 at.% to 3.9 at.%, respectively. This result is in good agreement with the FTIR spectra, where the degree of imidization was observed to increase with the curing temperature (Figure 1a).

The deconvoluted O 1s spectra are shown in Figure 2b. The peaks at 531.8 eV and 533.3 eV are attributed to C=O and C–O bonding in the PMDA-ODA structure, respectively. Overall, oxygen concentration related to PMDA-ODA decreased from 22.1 atomic % O at 200 °C to 17.5 atomic % O at 380 °C, indicative of the removal of O as the degree of imidization increased. 

**Figure 3:**
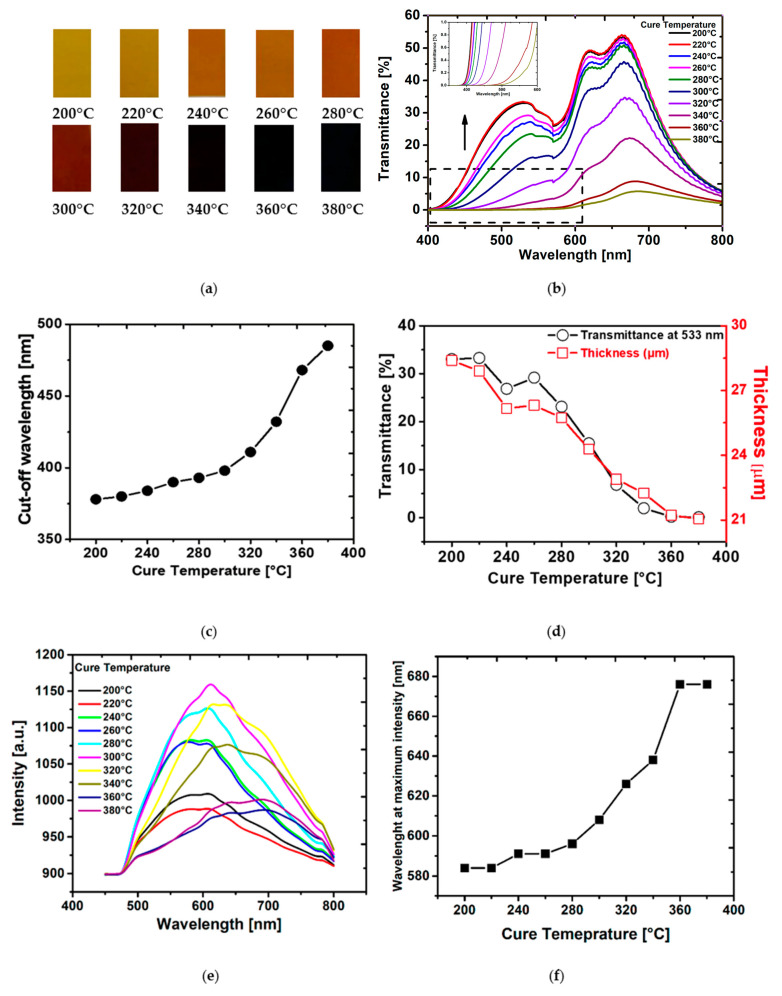
(**a**) Color shade of the PI films baked at different temperatures. (**b**) Transmittance in UV–vis spectroscopy of the PI films cured at different temperatures. (**c**) Cut-off wavelength for polyimide films. (**d**) Comparison between the transmittance at 533 nm with the PI films thickness changes as a function of cure temperature. (**e**) Steady-state photoluminescence spectra with excitation wavelength of 405 nm observed for PI films thermally cured at different temperatures. (**f**) Effect of cure temperature on the red-shift of the fluorescence spectrum of PMDA-ODA.

To demonstrate the effect that cure temperature has on the physical appearance of the PI, a photograph of the PI films cured at each temperature is presented in Figure 3a. It was noted that the films appear darker in color compared to the commercially available Kapton films. It is known that the use of NMP as a solvent leads to darker PI films when compared to using DMAc or DMF [34]. This is most likely related to trace amounts of the oxidized solvent remaining in the film. As NMP was used in this study, this may be a contributing factor to making the films appear darker than commercially available films.

It is obvious from Figure 3a that there is a progressive darkening of the color shade of the films with increasing cure temperature going from pale yellow to black [35,36]. To quantify the color change, a histogram intensity of the grey values (the brightness) of a pixel of each film was calculated and displayed using ImageJ software (Figure S6, see Supplementary Materials) demonstrating that the intensity decreases with increasing temperature. It is posited that the darkening of the films with cure temperature is primarily due to enhanced CTC formation. Another factor that may have an effect is the presence of impurities (e.g., oxidized non-reacted diamines) [34]; however, for wholly aromatic polyimides such as PMDA-ODA, it is widely regarded that the coloration is predominantly related to increases in charge transfer interactions [37,38,39,40,41,42,43]. Since CTC formation increases with curing temperature, the color intensity increases concomitantly [44].

Spectroscopy has been used by researchers to track the optical properties of polyimide samples [45,46,47]. UV–Vis spectroscopy was employed here to determine the effect cure temperature has on the extent of CTC formation [48]. The UV–Vis spectra of the PI films cured at each temperature are shown in Figure 3b.

The transmittance decreases with increasing imidization temperature. PI films cured at 360 °C and 380 °C show lowest transmission in the wavelength range between 400 nm and 800 nm. As the CTC absorbs light [49], a low transmittance can be indicative of a high degree of CTC formation in the PI sample [50]. A high-cured temperature of the PI films is effective in increasing the extent of charge transfer complex formation between polyimide chains leading to a decrease in the intermolecular distance. The increased presence of oxidized impurities may also contribute to the reduction in transmittance as these will increase with curing temperature.

The cut-off wavelength is defined as the wavelength at which the transmittance is lower than 1% [51]. Figure 3c shows the plot of the cut-off wavelength as a function of curing temperature, the value of cut-off wavelength gradually increases from 378 to 485 nm with increasing the curing temperature from 200 °C to 380 °C. Since the CT interactions originates from the formation of charge transfer complex between alternating electron-donor (diamine) and electron-acceptor (dianhydride) (Figure S7, in the Supplementary Materials), PI cured at 380 °C will exhibit more CTC than film cured at 200 °C [52].

Mechanical profilometry was performed to analyze the extent to which the thickness of the PI film is affected by the cure temperature (Figure 3d) [53]. The thickness of the PI films decreased from 28.4 to 21.1 µm when increasing the curing temperature from 200 to 380 °C, respectively. This corresponds to an overall shrinkage of 26% over the whole temperature range. It is posited that CTC formation plays an important role in this effect as the interaction between electron donor (diamine) and electron acceptor (dianhydride) decreases the intermolecular distance and promotes closer packing of the polymer chains. This interaction results in a decrease in the final thickness of film.

Figure 3e shows the changes of the photoluminescence (PL) spectra vs. the curing temperature. The PL spectra of the film reveal a peak at 600 nm that increases simultaneously with curing temperature, showing that the formation of CTC structure occurs simultaneously with the progress of the imidization reaction [54,55,56]. A maximum intensity was observed for films cured between 300 °C and 320 °C corresponding to the maximum degree of imidization, a decrease in the intensity appears above this temperature, and could be explained by the formation of some quenchers at high temperature [55]. The plot of wavelength at maximum intensity vs. curing temperature exhibits a red shift with increasing curing temperature (Figure 3f). As described by Donghwan et al. in their study on the effect thermal imidization and curing has on the fluorescence behavior of a phenylethynyl-terminated poly(amic acid) [57], this red shift is most likely related to a decrease in the intermolecular distance due to enhanced CTC formation in the polymers cured at higher temperatures. This is in agreement with the conclusion we drew from the optical properties and UV–Vis spectra of the films investigated in this study.

**Figure 4:**
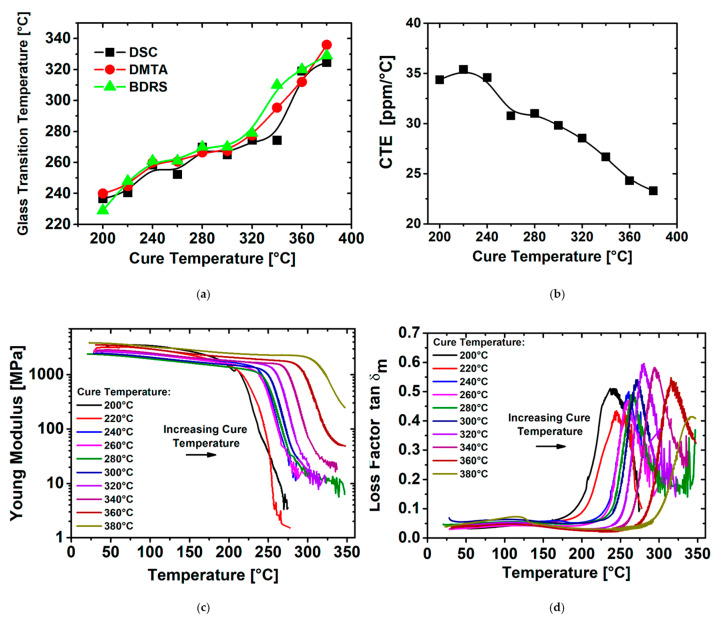
(**a**) Comparison between T_g_ obtained from DSC, DMTA (1 Hz) and BDRS (1 kHz). (**b**) CTE versus the cure temperature. (**c**) Mechanical storage modulus and (**d**) mechanical loss factor as a function of the measurement temperature and for the different cured films.

Thermal properties are important for PI films as they are potentially used as high-performance and heat-resistant engineering plastics. DSC, DMTA and BDRS analysis was performed to study the thermal behavior of the films cured at each temperature. Figure 4a shows the T_g_ for each sample as a function of curing temperature, calculated from the DSC curves (Figure S8, see Supplementary Materials), DMTA and BDRS. It was observed that the T_g_ shifted to higher temperatures with increased curing temperature. The value of T_g_ depends on the mobility of the polyimide chains and is dependent on the cooperative macroscopic macromolecular movements when the film enters the viscoelastic range. When used in a device, a higher T_g_ facilitates a wider operating temperature range. The increase in T_g_ observed is related to the increase in the extent of the CTC formation as this progressively increases the interchain interaction. This results in higher energy necessary for rotation and a higher heat capacity, and thus, higher T_g_ values [58,59]. In addition to this, we observed a similarity in shape between the cut-off wavelength (Figure 3c) and T_g_ (Figure 4a) for polyimide films, both increasing with curing temperature; this confirms that the improvement in glass temperature is the result of CTC formation.

Figure 4b shows the effect of PI curing temperature on the CTE. The CTE decreases markedly when the curing temperature increases, dropping from 34.4 ppm/°C to 23.3 ppm/°C when cured from 200 °C up to 380 °C. This trend is in close agreement with the thickness changes depicted in Figure 4c and is a result of the denser molecular packing in the film [43].

DMTA measurements were used to characterize the thermo-mechanical properties of PI films (Young’s modulus E’ and loss factor tanδ_m_ for the different curing temperature conditions [60]. The maximum temperature prior to the sharp decrease in the Young’s storage modulus E’ corresponds to DMTA T_g_ values in Figure 4a [61,62]. Further analysis of the DMTA curves in Figure 4c shows that the magnitude of E’ gradually increases from 2.17 GPa to 4 GPa at room temperature with increasing PI curing temperature. In addition, the storage modulus exhibits a significant extension of the glassy plateau for the PI films cured at higher temperatures. Simultaneously, the plot of the mechanical loss factor, tanδ_m_, as a function of temperature shows a shift in the peak maxima with increasing PI cure temperature related to a shift in T_g_ towards higher temperature (Figure 4d). This is partly due to an increase in DOI [16], with the increase in PI chain packing density being the major contributing factor. This means that the mobility of the PI chains is restricted due to the interaction of adjacent molecules derived from the imidization process. It is worth noting that T_g_ values determined from both tanδ_m_ and through DSC measurements correlate quite well with each other (Figure 4a).

Furthermore, the magnitude of the mechanical tanδ_m_ indicates the energy dissipation of a material during cyclic stress in DMTA testing. This is an indicator of a material’s ability to absorb energy. A material is perfectly elastic if tanδ_m_ = 0, perfectly viscous if tanδ_m_ is infinite and equally elastic and viscous when tanδ_m_ = 1. The tanδ_m_ of the various PI films reaches a peak maximum around 0.59 at 320 °C. It suggests that the thermal imidization process increases the mechanical rigidity of the PI films. This restricts free movements of the molecular chains and makes them behave less elastically [63]. The previous observation of lower CTE values for the higher cured PI films are clearly related here to the higher values of E’ in the rubbery state. Finally, it was observed for the films with a higher DOI that a higher thermal energy is required to develop the necessary macromolecular mobility to detect T_g_.

**Figure 5:**
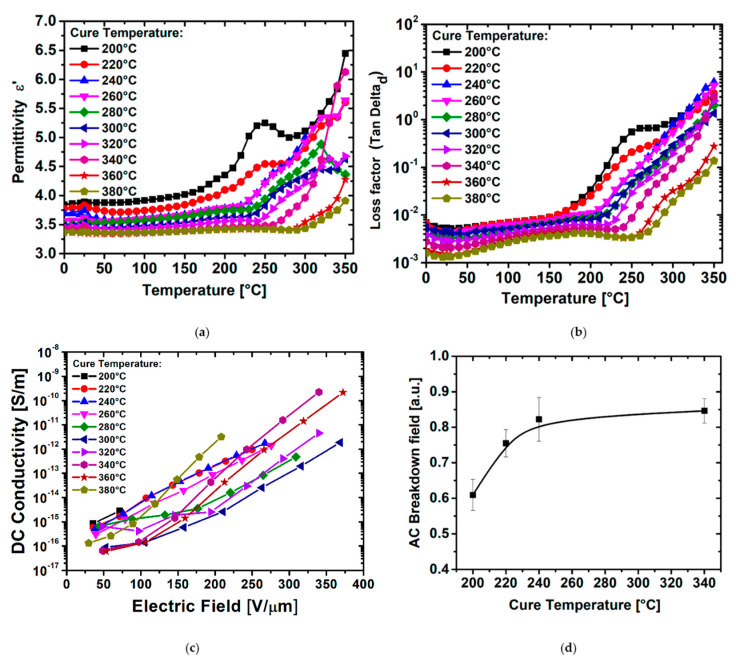
(**a**) Dielectric permittivity and (**b**) dielectric loss factor at 1 kHz vs. temperature. (**c**) DC conductivity vs. electric field for different PI imidization temperatures. (**d**) Dielectric breakdown field of PI films vs. imidization temperature.

The dielectric and electrical properties of PI films, such as for the mechanical, optical, and thermal properties, are directly related to the chemical structure of the PI backbone. From the previous characterization, one could expect that PI films cured at the highest temperature should present fewer losses and a higher breakdown threshold due to the lower impurity density in bulk (e.g., less ionizable COOH groups releasing free H^+^ protons) and higher chain packing. Figure 5 shows the temperature dependence of both the permittivity (a) and dielectric factor loss tanδ_d_ (b) for the different PI films measured at 1 kHz. The permittivity ε’ and loss factor tanδ_d_ present a first relaxation phenomenon occurring between 0 and 150 °C well-known as the β-relaxation [64]. The β-relaxation corresponds to a sub-glass transition local dipolar relaxation mode due to imide group movements coming either from the dianhydride moiety or to rotations of the para-phenylene moieties in the diamine part. It is noteworthy that the increase in the PI cure temperature gives a mitigation of the β-relaxation magnitude. Moreover, a second relaxation of higher magnitude, that occurs above 170 °C and is a result of a combination between the α-relaxation and the charge conduction due to T_g_, appeared to be widely affected by the PI curing temperature. This relaxation, on both ε’ and tanδ_d_, remarkedly shifts to a higher temperature when the PI curing temperature increases from 200 °C up to 380 °C.

Higher electric field characterizations were used to determine the electrical properties when the PI films are highly stressed closer to real applicative operating conditions. Consequently, CC measurements have been performed up to 350 V/µm to extract the field-dependency of the DC conductivity σ_DC_, as shown in Figure 5c. The conductivity σ_DC_ is poorly dispersed with very low values between 10^−16^ and 10^−15^ Ω^−1^ m^−1^ at low field up to 100 V/µm whatever the curing temperature confirming the dielectric properties. At higher field, σ_DC_ shows a slight divergence between the different curing conditions. The charge density (and/or their mobility) can be strongly reduced by increasing imidization temperature for PMDA/ODA PI films. Earlier, Diaham et al. have reported on a 400 °C optimal imidization temperature in the case of BPDA/PDA PI [65], where CTC has less effect. In the present PMDA/ODA PI films, from 340 °C the conductivity increases again (Figure 5c). This tendency highlights a detrimental effect of the PI chain packing on the high electric field characteristics. XRD analysis revealed that the films have a low degree of crystallinity (Figure S9), demonstrating that the higher chain packing density in the PMDA/ODA PI films does not result in a crystallization of the molecular structure. Therefore, the higher chain packing density does not bring similar beneficial electrical effects such as in semicrystalline polymers [66,67]. The CTC formation could give rise to an extension of charge density in the PI bulk when cured at too high a temperature. Such a drawback can be easily mitigated when the PI films are cured at a lower temperature. On the contrary, an under-cure of PI films favors a non-complete imidization process that leads to the residual presence of ionizable PAA impurities and giving high conductivity values under high field.

Finally, the breakdown field is one of the most important parameters that characterizes the electrical properties of insulating materials. The changes in the AC dielectric breakdown field (E_BR_) as a function of the imidization temperature are shown in Figure 5d. The evolution of E_BR_ with the curing temperature clearly reveals a continuous improvement going to higher curing temperature. At lower curing temperatures, the breakdown field collapses due to a low DOI.

## 4. Summary of the Thermal Imidization Process

Over the course of this study, it has been shown that the curing temperature has a major impact on the final properties of polyimide films. FTIR analysis has shown that the DOI of PI increases from 200 °C before reaching a maximum at 320 °C (Figure 1c), beyond which it plateaus. Another phenomenon takes place during the thermal imidization. Indeed, the charge–transfer complex formation, which is an association of two or more molecules, or of different parts of one large molecule, in which a fraction of electronic charge is transferred between the molecular entities (see Supplementary Figure S7).

A high cured temperature of PI films is effective in increasing the CTC (Figure 3c) between polyimide backbone chains through steric hindrance. This leads to decrease in the intermolecular distance and thus increased the interaction between the PI chains resulting in a good packing and less free volume which affect the final polyimide thickness (Figure 3d). As the CTC absorbs light, the PI color becomes darker during the thermal curing (Figure 3a). On the other hand, as the cure temperature varies, the strength of the CTC increases and involves an enhancement in glass transition temperature during the thermal imidization (Figure 4a) representing a difference in intermolecular chain interactions. This results in higher energy necessary for rotation, higher heat capacity and higher T_g_.

While the mechanical (Figure 4c,d) breakdown field and dielectric properties (Figure 5a,b,d) are continuously enhanced with cure temperature increase, the field-dependent electrical properties present an optimized imidization within the intermediate temperature range (Figure 5c). The electrical characterization at low field presents an enhancement of the final PI properties likely due to a DOI extent. On the contrary, at high electric field, the conductivity results show an improvement in the cure temperature at an intermediate temperature emphasizing an ideal compromise between a high DOI and a contained PI chain packing when the thermal imidization process is performed over this equilibrium.

**Figure 6:**
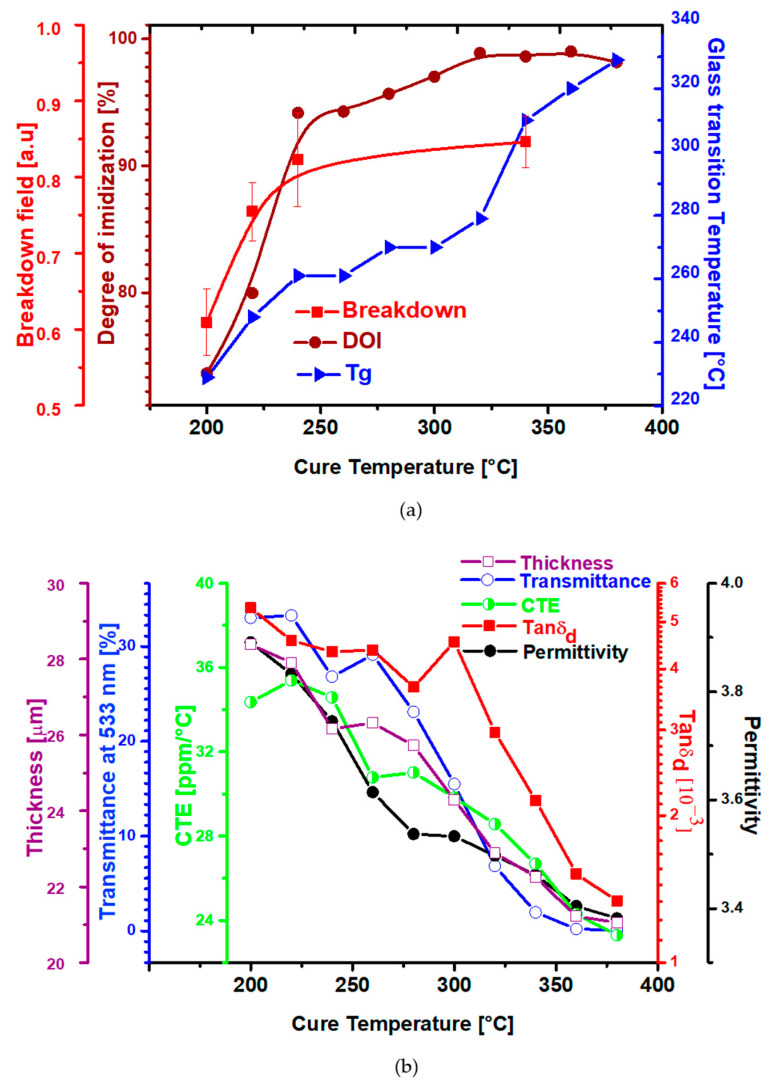
Universal plot of the main physical changes in PMDA-ODA polyimide films over the thermal imidization process: (**a**) DOI, T_g_, E_BR_, and (**b**) thickness, UV–vis transmittance, CTE, tanδ_d_, and ɛ’.

Figure 6 summarizes in a universal plot of the main changes that occur in PI films over the thermal imidization process.

## 5. Conclusions

This paper reports the widest ranging investigation published to date on the effect that thermal imidization has on the properties of polyimide (specifically PMDA-ODA). A complete cross-analysis of the main physical and chemical properties was performed as a function of the curing temperature via in-depth physico-chemical, thermal, mechanical and electrical characterisation. The analysis revealed that the degree of imidization increased from 200 °C up to 320 °C, reaching a plateau beyond that. UV-Vis and photoluminescence spectroscopy revealed that the extent of charge transfer complex formation increased over the entire curing temperature range (i.e., 200 °C up to 380 °C). Electrical characterization revealed that the dielectric properties are enhanced with increasing cure temperature, while the field-dependent electrical properties are optimal in the intermediate temperature range. This is due to a compromise in performance related to maximizing the degree of imidization (i.e., mitigation of ionizable impurities) and the optimization of the degree of charge–transfer complex formation (i.e., mitigation of the chain packing). This balance enables the maximum performance of the PI film to be obtained with respect to the optimal electrical, thermal and mechanical properties by controlling the cure temperature.

## Figures and Tables

**Table 1:** State-of-the-art of previous studies on the imidization temperature effects on polyimide physico-chemical, thermal, mechanical and electrical properties with respect to the present work. The ‘+’ means that the technique has been used in the referenced studies.

			Main Polyimide Properties													
			Physico-Chemical				Thermal		Mechanical				Electrical			
Polyimide Type	Imidization Curing (°C)	Process	FTIR	XRD	XPS	UV–Vis	TGA (T_d_)	DSC (T_g_)	DMTA (E’, E”, tanδ_m_)	Tensile Strength and Elongation	CTE	Thickness	BDRS (ε’, ε”, tanδ_d_, σ_AC_)	CC (σ_DC_)	BD (E_BR_)	References
PMDA-ODA	250–400	Spin-coat		+												[8]
PMDA-ODA	97–447	VDP			+											[9]
PMDA-ODA	150 vs. time	VDP	+											+		[10]
PMDA-ODA	85–400	Spin-coat	+				+	+								[11]
PMDA-ODA	135–350	Spin-coat	+									+				[12]
PMDA-ODA	200–350	VDP	+				+	+								[13]
PMDA-ODA	170–350	VDP	+													[14]
PMDA-ODA	100–500	Spin-coat	+									+				[15]
PMDA-ODA PMDA-PDA	100–400	Casting	+	+							+					[16]
PMDA-ODA	100–400	Spin-coat	+						+			+				[17]
PMDA-ODA	300 vs. time	VDP	+						+	+						[18]
PMDA-ODA	150–350	VDP	+				+						+	+	+	[19]
PMDA-ODA	170–350	VDP	+				+									[20]
PMDA-ODA	80–300	VDP	+				+									[21]
PMDA-ODA	30–380	Spin-coat	+	+				+								[22]
PMDA-ODA	70–400	Casting	+	+			+	+	+	+						[23]
PMDA-BDA	100–250	Casting		+			+	+	+	+						[24]
BPDA-ODA ODPA-ODA	180–380	Casting	+						+	+						[25]
BPDA-PDA BDPA-ODA	200–400	Dr.-blade		+		+	+	+	+		+	+				[26]
BPDA-ODA-PDA	200–350	Spin-coat	+				+			+	+	+	+	+	+	[27]
BTDA-ODA-MPDA	125–400	Spin-coat	+									+				[28]
BTDA-type	25–300	N/A	+				+					+			+	[29]
PMDA-BACB	140–250	N/A	+		+		+	+								[30]
BPDA-PDA	175–450	Spin-coat	+				+	+					+	+	+	[31]
**PMDA-ODA**	**200–380**	**Spin-coat**	**+**	**+**	**+**	**+**	**+**	**+**	**+**	**+**	**+**	**+**	**+**	**+**	**+**	**This work**

## Data Availability

Not applicable.

## Supplementary Materials

The following supporting information can be downloaded at: https://www.mdpi.com/article/10.3390/polym14091713/s1, Figure S1: TGA curves of PI film cured at 200 °C; Figure S2: Effect of curing temperature on the FTIR spectra; Figure S3: DOI on each side of the PI films; Figure S4: FTIR spectra of uncured Polyimide; Figure S5: High resolution XPS scans of N; Figure S6: Histogram of intensity; Figure S7: Charge transfer complex sketch describing the packing of the PI chains; Figure S8: DSC curves of PI cured at (a) 200°Cm (b) 220°C, (C) 240°C, (d) 260°C, (e) 280°C, (f) 300°C, (g) 320°C, (h) 340°C, (i) 360°C and (j) 380°C; Figure S9: XRD patterns of PIs films with different cure temperature.
