# Supplementary material for: A Universal Study on the Effect Thermal Imidization Has on the Physico-Chemical, Mechanical, Thermal and Electrical Properties of Polyimide for Integrated Electronics Applications
Source: Polymers (Basel). 2022 Apr 22;14(9):1713. doi: 10.3390/polym14091713 (PMC9101791; doi:10.3390/polym14091713)
Supplement: Supplementary file 1 [file polymers-14-01713-s001.zip › polymers-1608306-supplementary.pdf]

Supporting information : A Universal Study on the Effect Thermal Imidization Has on the Physico-Chemical, Mechanical, Thermal and Electrical Properties of Polyimide for Integrated Electronics Applications

Imadeddine Benfridja [1,2,3], Sombel Diaham [3], Fathima Laffir [2], Grace Brennan [2,4], Ning Liu [2,4] and Tadhg Kennedy[1,2]

[1] Department of Chemical Sciences, University of Limerick, Limerick V94 T9PX, Ireland; imadeddine.benfridja@ul.ie
[2] Bernal Institute, University of Limerick, Limerick V94 T9PX, Ireland; fathima.laffir@ul.ie (F.L.), gracebrennan11@gmail.com (G.B.), ning.liu@ul.ie (N.L.)
[3] LAPLACE Institute, University of Toulouse, Université Paul Sabatier, Toulouse 31062, France; sombel.diaham@laplace.univ-tlse.fr
[4] Department of Physics, University of Limerick, Limerick V94 T9PX, Ireland
* Correspondence: tadhg.kennedy@ul.ie

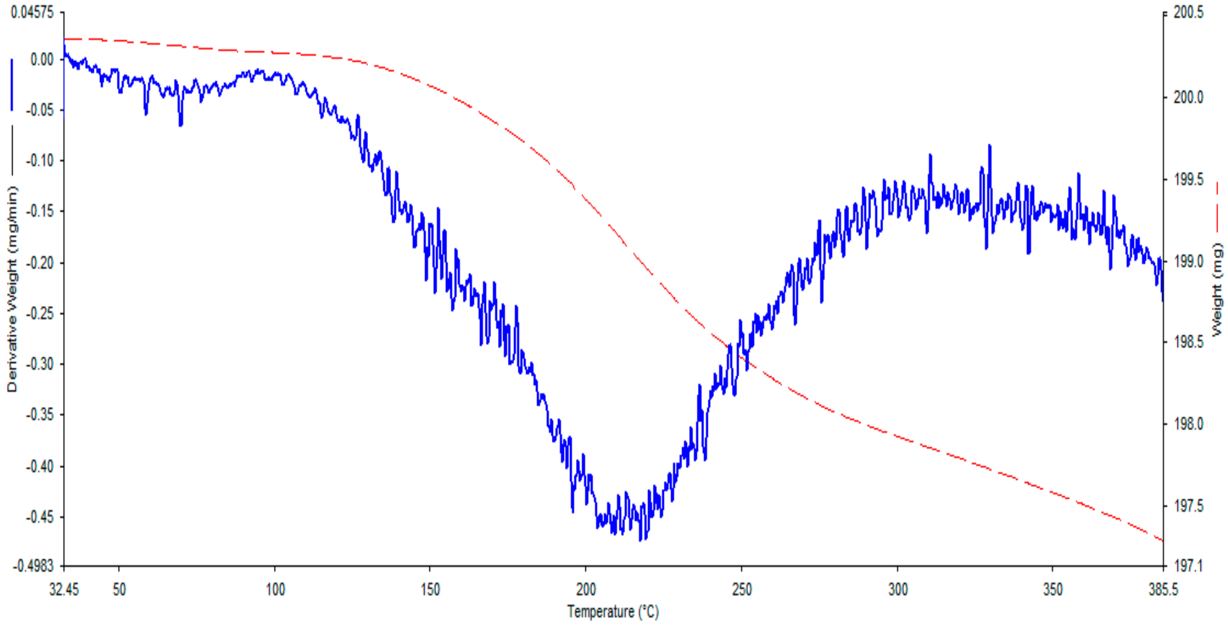

Figure S1. TGA curves of PI film cured at 200°C.

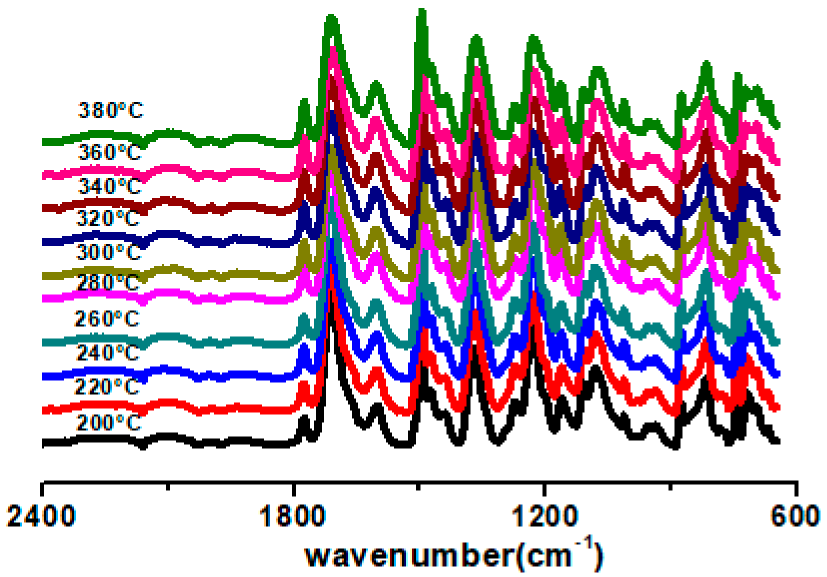

Figure S2. Effect of curing temperature on the FTIR spectra

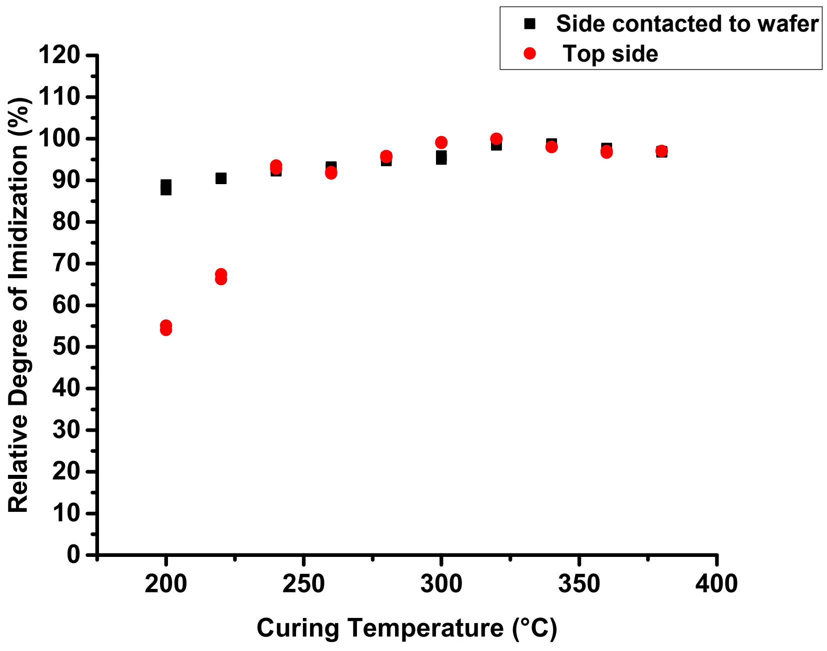

Figure S3. DOI on each side of the PI films.

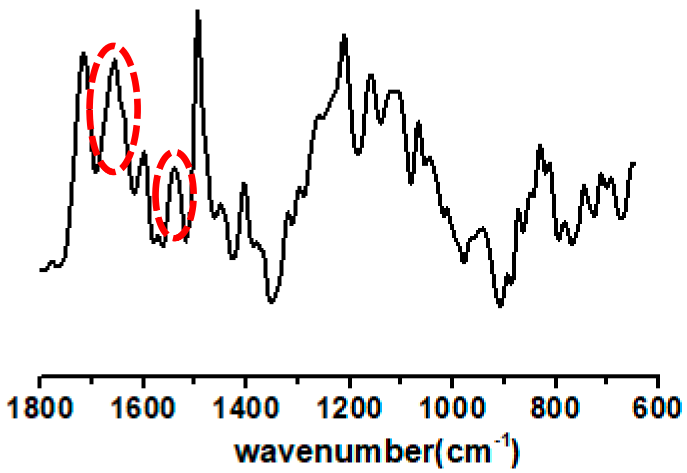

Figure S4. FTIR spectra of uncured Polyimide

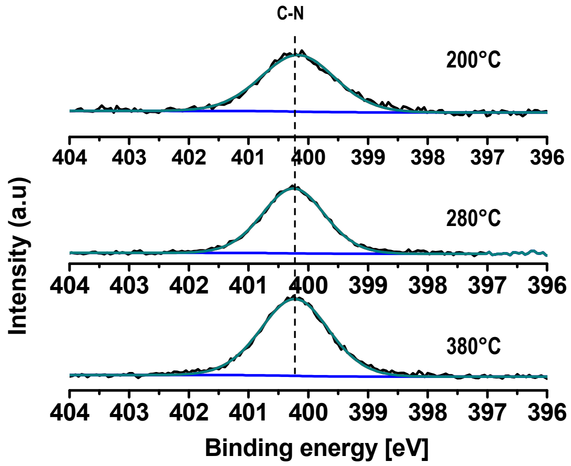

Figure S5. High resolution XPS scans of N.

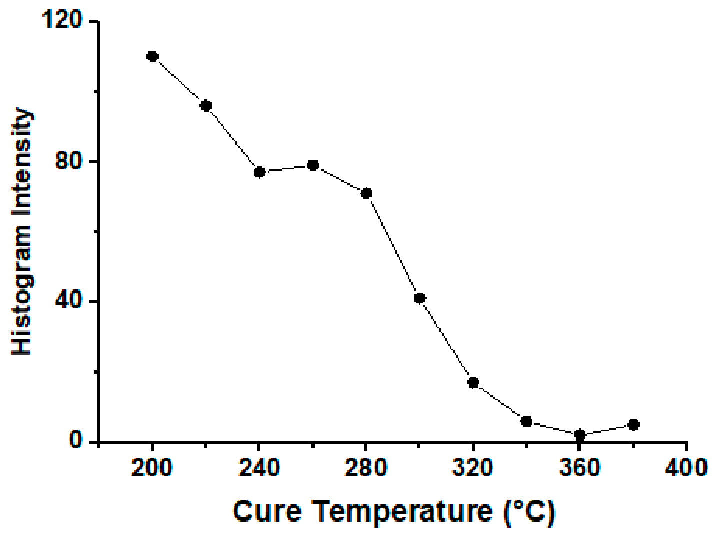

Figure S6. Histogram of intensity.

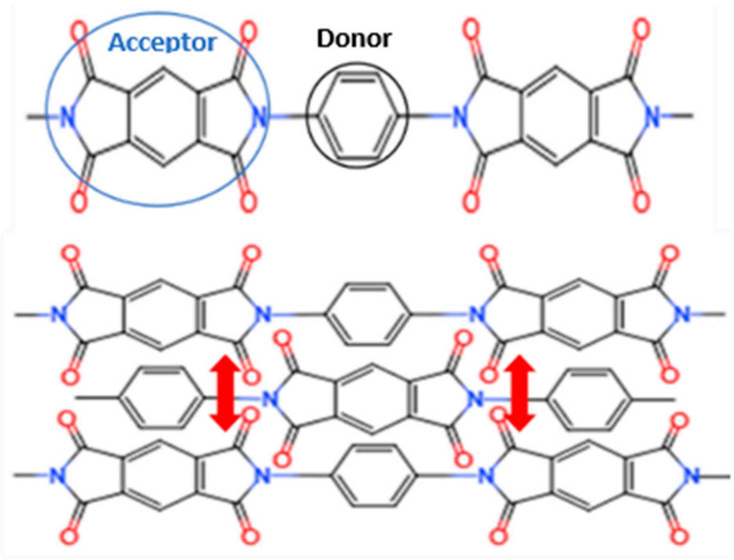

Figure S7. Charge transfer complex sketch describing the packing of the PI chains

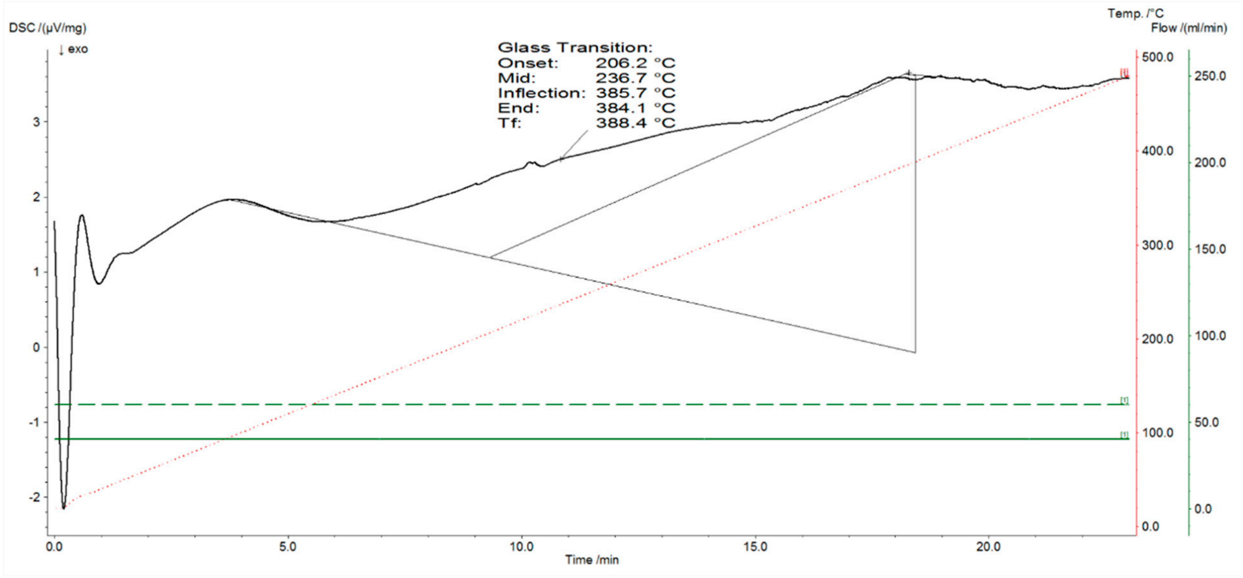

DSC /(µV/mg)
↓ exo
Glass Transition:
Onset: 206.2 °C
Mid: 236.7 °C
Inflection: 385.7 °C
End: 384.1 °C
Tf: 388.4 °C
Temp. /°C
Flow /(ml/min)
Time /min

(a)

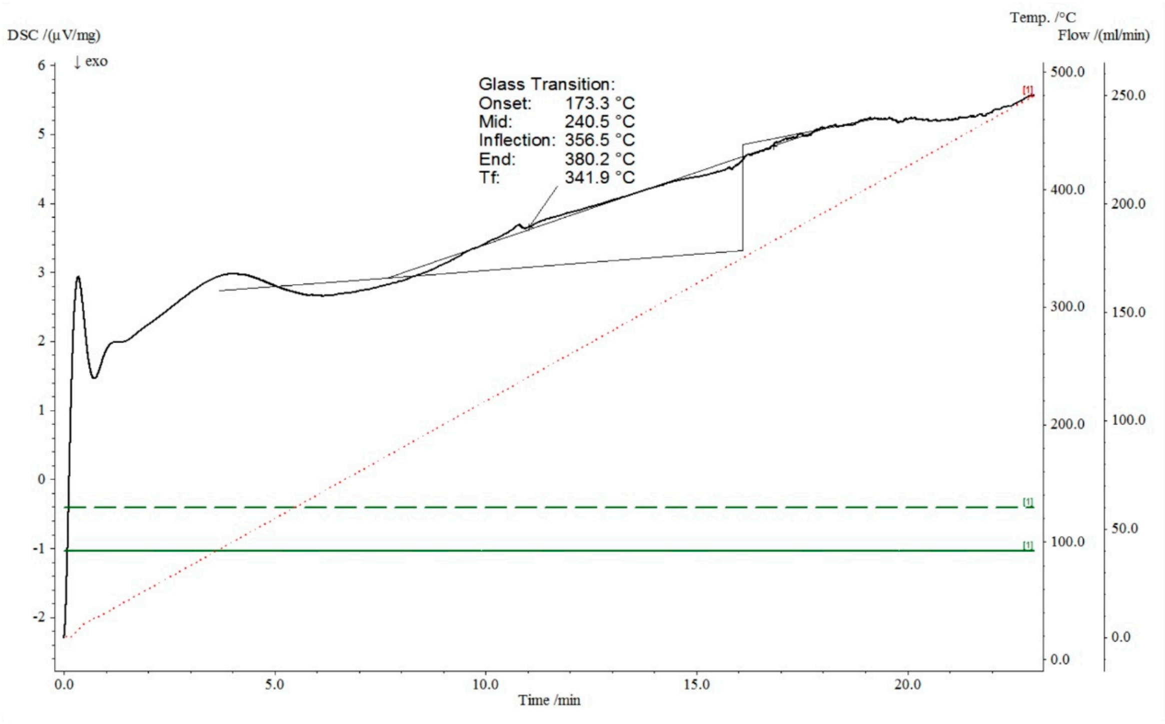

DSC /(µV/mg)
↓ exo
Glass Transition:
Onset: 173.3 °C
Mid: 240.5 °C
Inflection: 356.5 °C
End: 380.2 °C
Tf: 341.9 °C
Temp. /°C
Flow /(ml/min)
Time /min

(b)

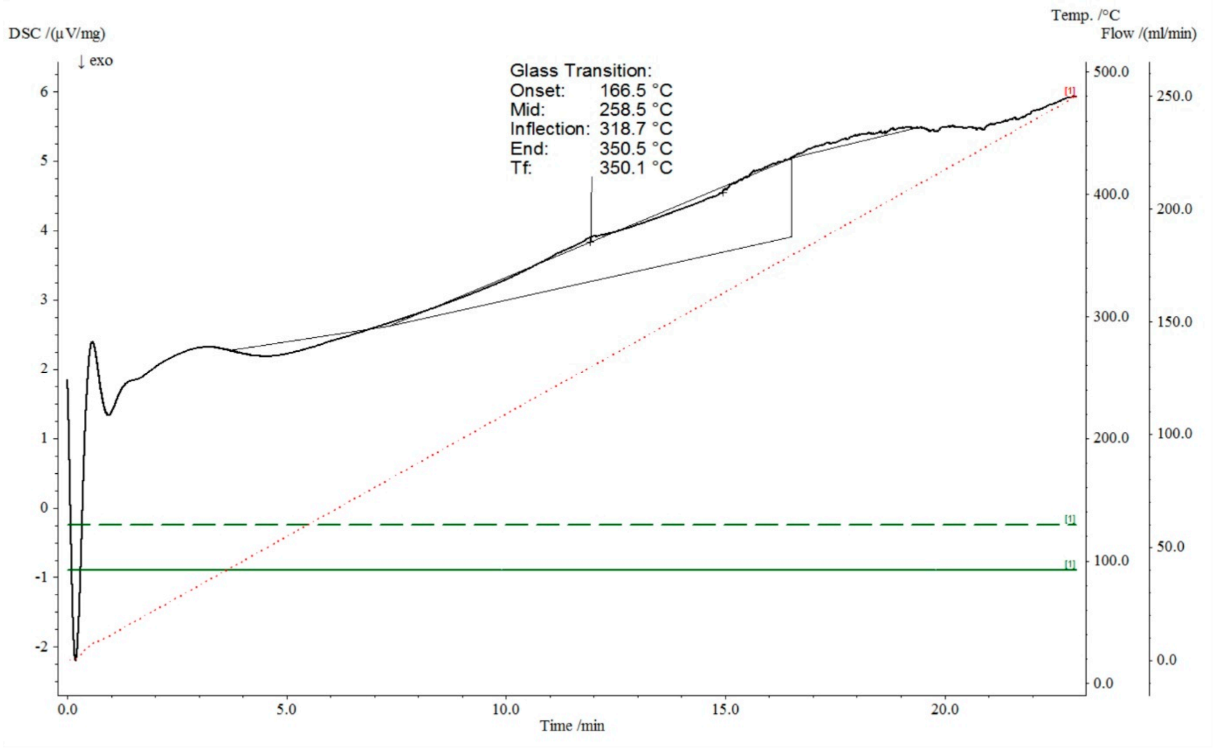
DSC /(μV/mg)
↓ exo
Temp. /°C
Flow /(ml/min)
Glass Transition:
Onset: 166.5 °C
Mid: 258.5 °C
Inflection: 318.7 °C
End: 350.5 °C
Tf: 350.1 °C
Time /min

(c)

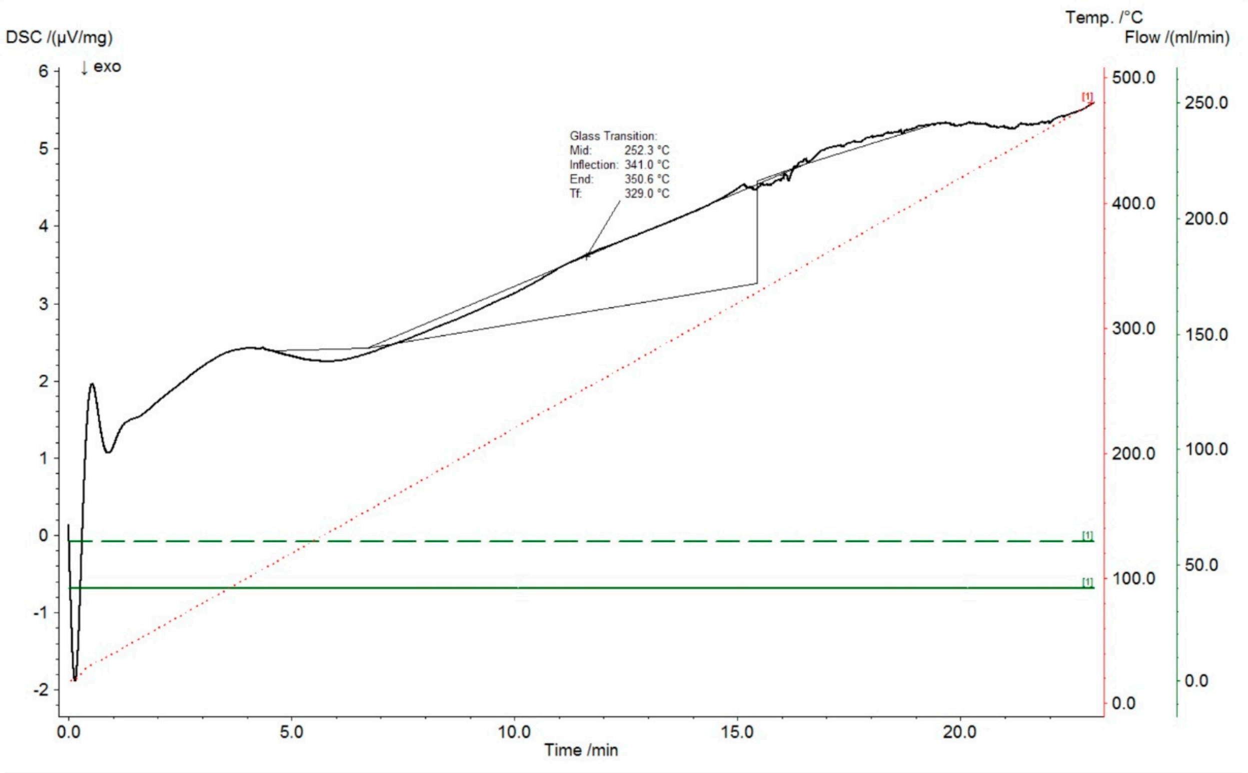
DSC /(μV/mg)
↓ exo
Temp. /°C
Flow /(ml/min)
Glass Transition:
Mid: 252.3 °C
Inflection: 341.0 °C
End: 350.6 °C
Tf: 329.0 °C
Time /min

(d)

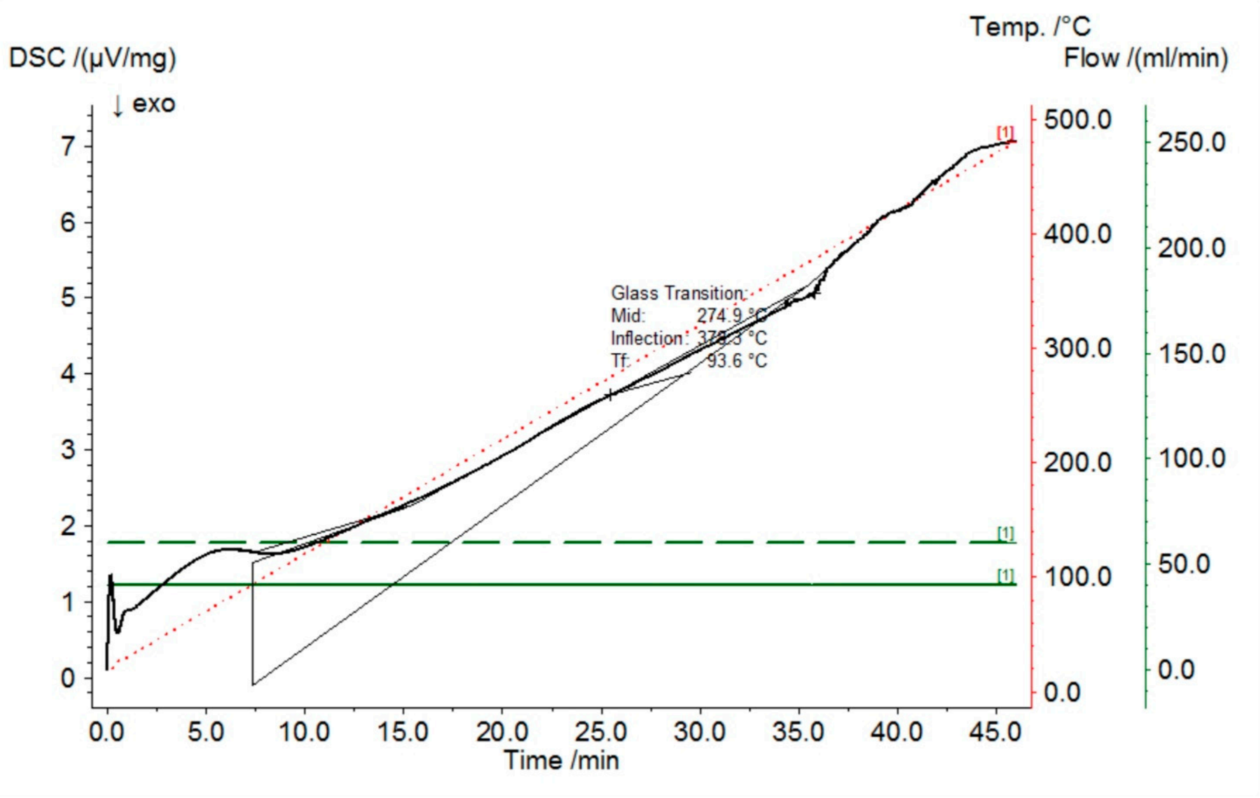

DSC /(µV/mg)
↓ exo
Temp. /°C
Flow /(ml/min)
Glass Transition:
Mid: 274.9 °C
Inflection:
Tf: 93.6 °C
[1]
[1]
[1]
0
1
2
3
4
5
6
7
0.0
100.0
200.0
300.0
400.0
500.0
0.0
50.0
100.0
150.0
200.0
250.0
0.0 5.0 10.0 15.0 20.0 25.0 30.0 35.0 40.0 45.0
Time /min

(e)

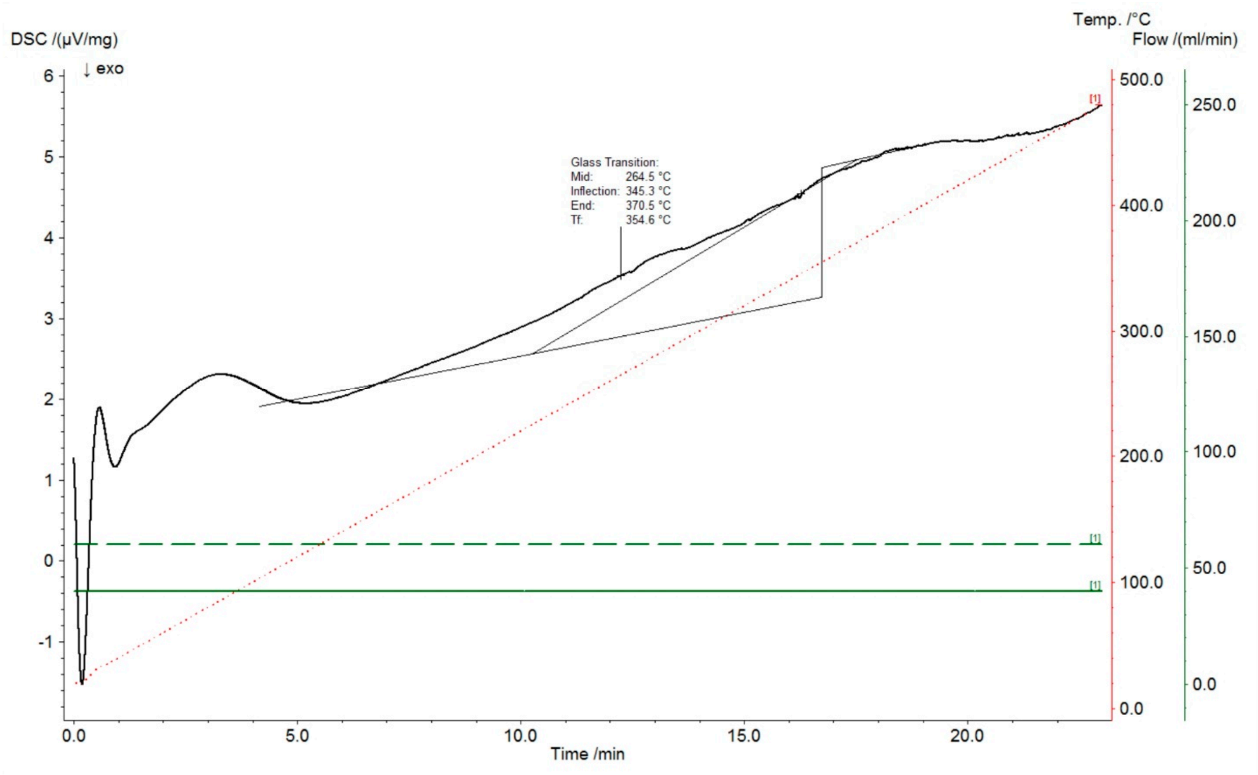

DSC /(µV/mg)
↓ exo
Temp. /°C
Flow /(ml/min)
Glass Transition:
Mid: 264.5 °C
Inflection: 345.3 °C
End: 370.5 °C
Tf: 354.6 °C
[1]
[1]
[1]
-1
0
1
2
3
4
5
6
0.0
100.0
200.0
300.0
400.0
500.0
0.0
50.0
100.0
150.0
200.0
250.0
0.0 5.0 10.0 15.0 20.0
Time /min

(f)

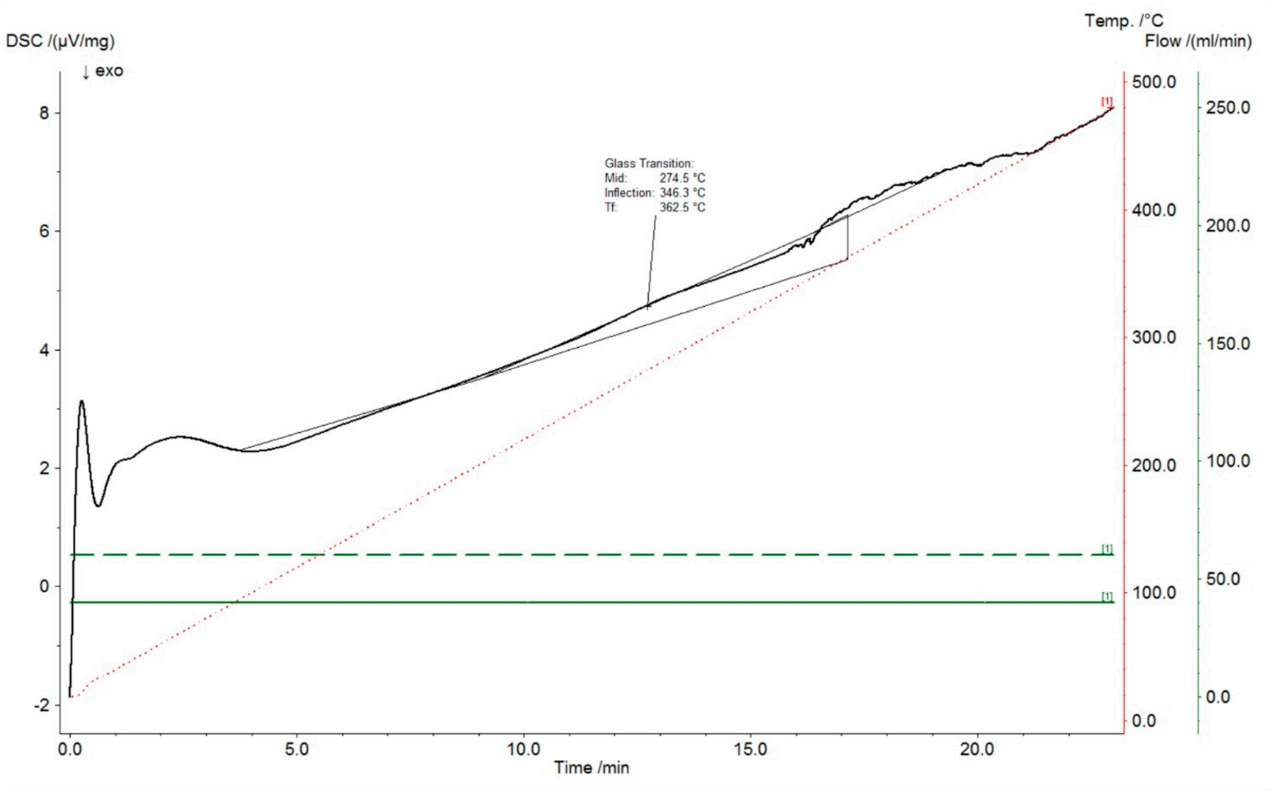
DSC /(µV/mg)
↓ exo
Temp. /°C
Flow /(ml/min)
Glass Transition:
Mid: 274.5 °C
Inflection: 346.3 °C
Tf: 362.5 °C
[1]
[1]
[1]
8
6
4
2
0
-2
500.0
400.0
300.0
200.0
100.0
0.0
250.0
200.0
150.0
100.0
50.0
0.0
0.0
5.0
10.0
15.0
20.0
Time /min

(g)

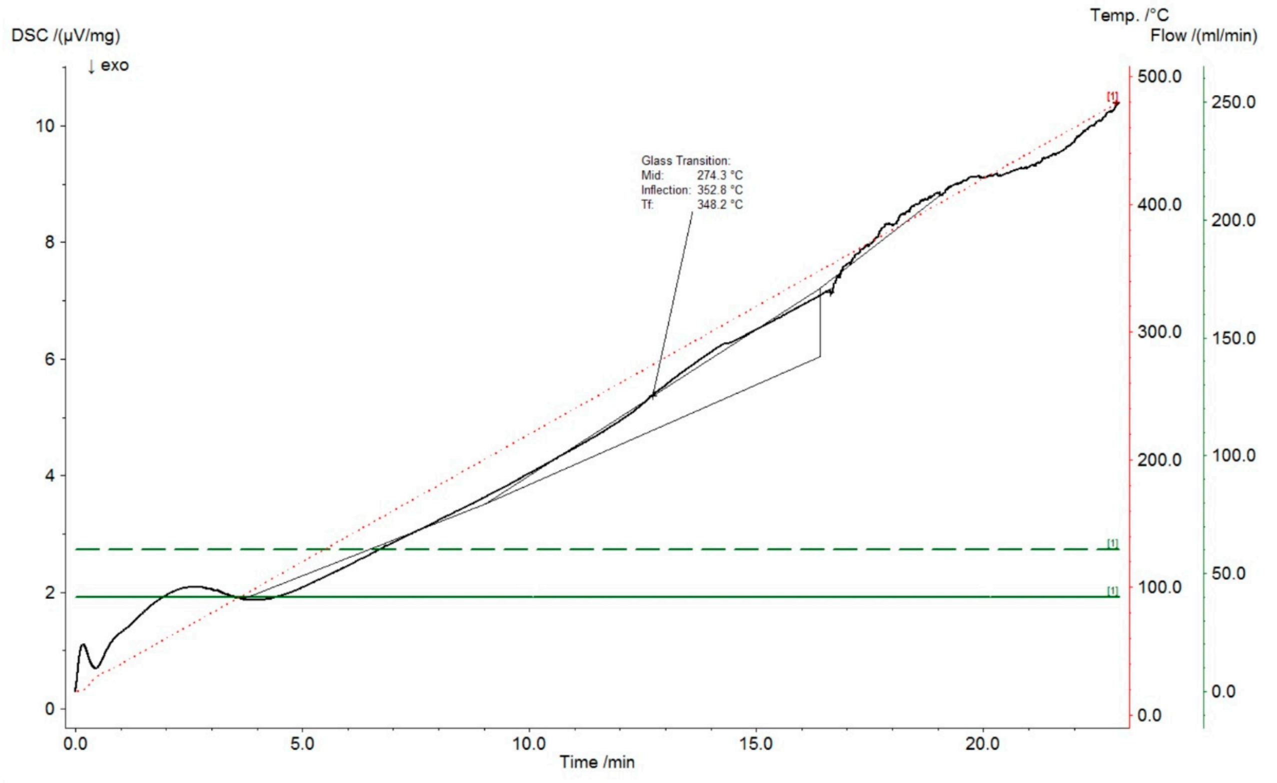
DSC /(µV/mg)
↓ exo
Temp. /°C
Flow /(ml/min)
Glass Transition:
Mid: 274.3 °C
Inflection: 352.8 °C
Tf: 348.2 °C
[1]
[1]
[1]
10
8
6
4
2
0
500.0
400.0
300.0
200.0
100.0
0.0
250.0
200.0
150.0
100.0
50.0
0.0
0.0
5.0
10.0
15.0
20.0
Time /min

(h)

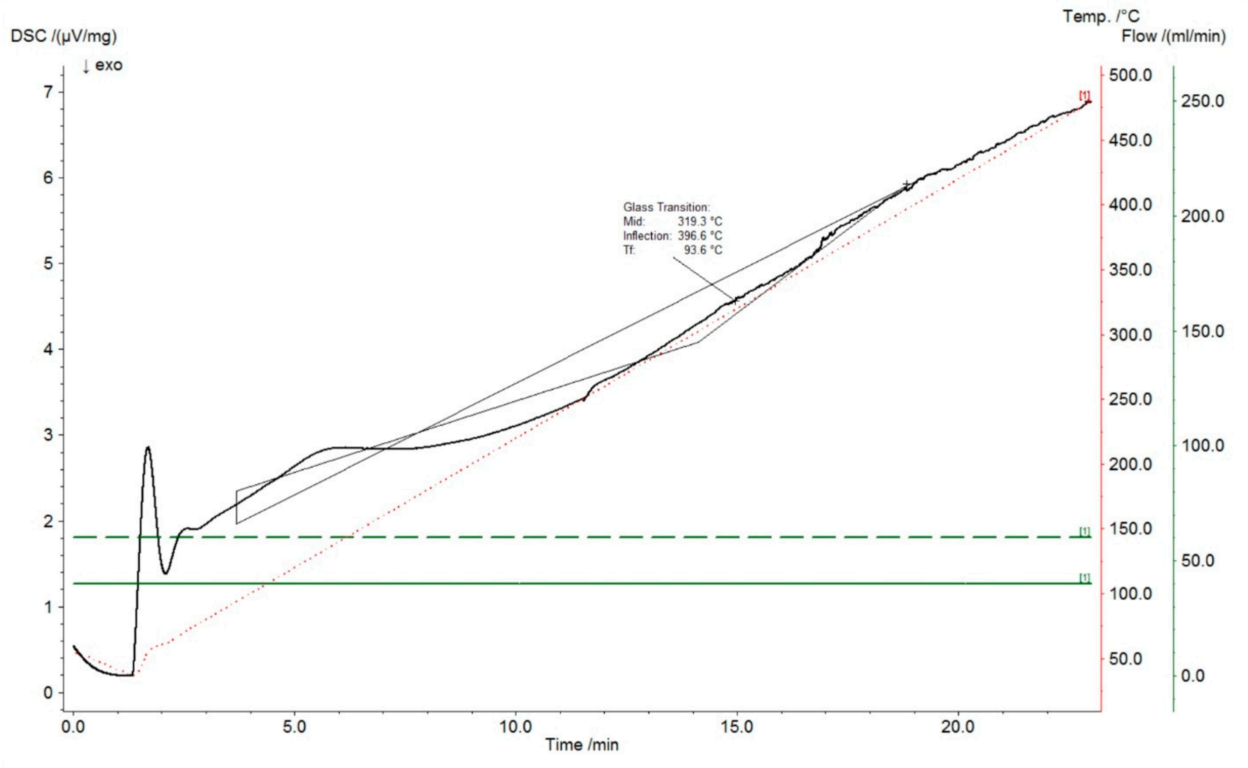

(i)

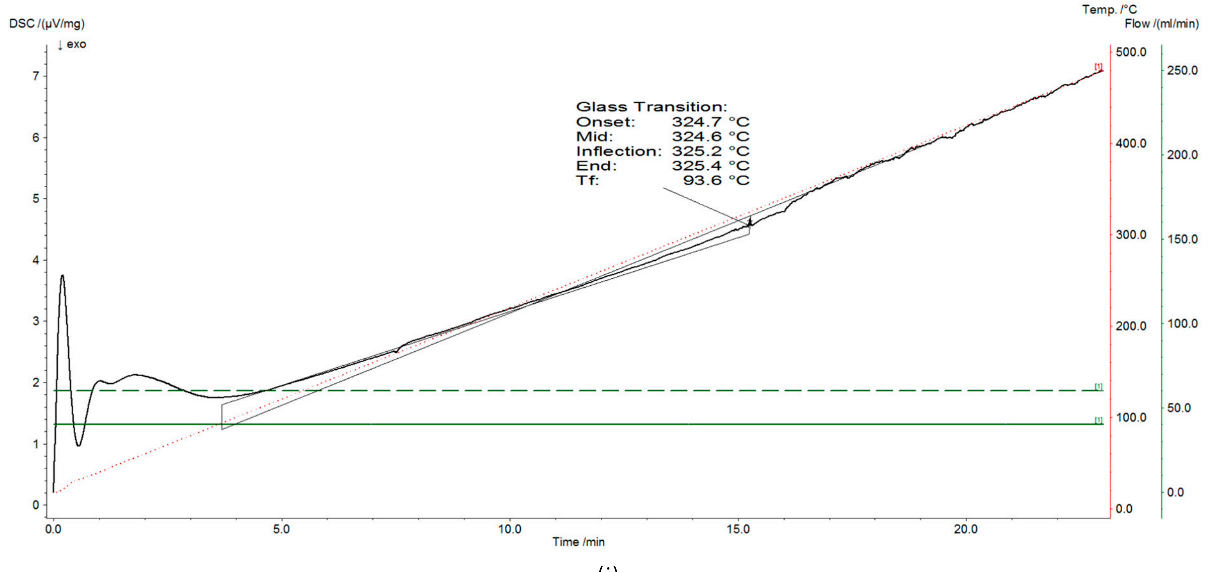

(j)

Figure S8. DSC curves of PI cured at (a) 200°Cm (b) 220°C, (C) 240°C, (d) 260°C, (e) 280°C, (f) 300°C, (g) 320°C, (h) 340°C, (i) 360°C and (j) 380°C.

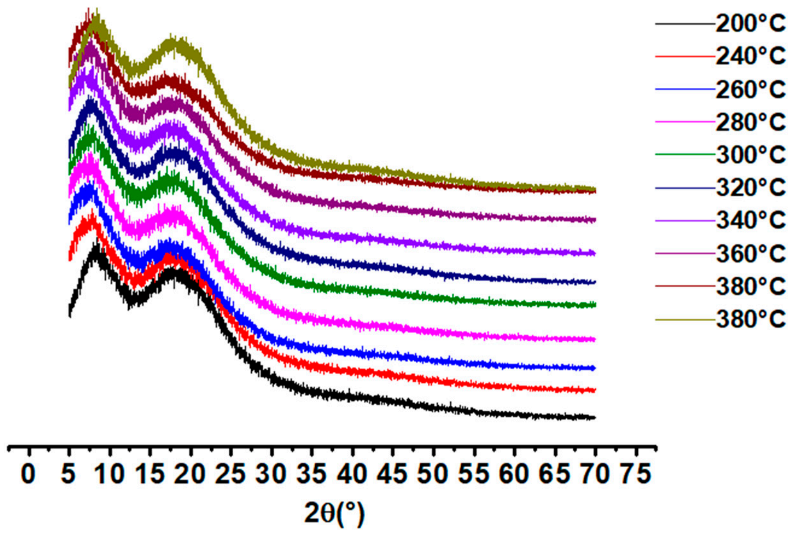

Figure S9. XRD patterns of PIs films with different cure temperature
